# Explainable neuro-symbolic artificial intelligence for automated interpretation of corneal topography and early keratoconus detection

**DOI:** 10.3389/frai.2026.1713747

**Published:** 2026-04-13

**Authors:** Mini Han Wang, Shuai Qin

**Affiliations:** 1 Zhuhai People’s Hospital (The Affiliated Hospital of Beijing Institute of Technology, Zhuhai Clinical Medical College of Jinan University), Zhuhai, Guangdong, China; 2 Zhuhai Institute of Advanced Technology Chinese Academy of Sciences, Zhuhai, Guangdong, China; 3 The Faculty of Medicine, The Chinese University of Hong Kong, Hong Kong, Hong Kong SAR, China; 4 Department of Ophthalmology, The Third Affiliated Hospital of Southern Medical University, Guangzhou, China

**Keywords:** corneal topography, explainable AI, keratoconus detection, knowledge graph, large language models, neuro-symbolic artificial intelligence, refractive surgery screening

## Abstract

**Background:**

Early detection of keratoconus is essential for preventing postoperative complications in refractive surgery and preserving long-term visual function. Although artificial intelligence has demonstrated strong potential in ophthalmic image analysis, many existing models operate as black-box systems and provide limited clinical interpretability. Transparent decision support is therefore critical for safe deployment of AI in clinical practice.

**Methods:**

We propose an explainable neuro-symbolic framework for automated interpretation of corneal topography reports and refractive surgery eligibility assessment. The proposed system integrates multimodal feature extraction, a symbolic corneal knowledge graph, probabilistic reasoning, and large language model (LLM)–based report generation. Quantitative biometric parameters and corneal curvature maps extracted from IOLMaster 700 reports were processed through a hybrid convolutional neural network–Vision Transformer (CNN–ViT) module to capture spatial corneal morphology. These representations were aligned with a clinically curated knowledge graph encoding relationships between corneal parameters, disease states, and surgical decision criteria. Bayesian probabilistic inference was then applied to estimate disease likelihoods, while an ensemble LLM module generated structured bilingual clinical reports explaining the reasoning process.

**Results:**

In a prospective pilot cohort of 20 eyes, the proposed framework demonstrated strong diagnostic performance for early keratoconus detection, achieving an area under the receiver operating characteristic curve (AUC) of approximately 0.95. Sensitivity and specificity remained high across decision thresholds, and the system achieved a balanced F1 score for refractive surgery eligibility classification. Expert evaluation indicated high interpretability and clinical usefulness of the generated reports. The end-to-end pipeline required approximately 95 ± 12 s per case, supporting near–real-time clinical decision support.

**Conclusion:**

The proposed neuro-symbolic framework combines deep representation learning, structured medical knowledge, and explainable language-based reporting to provide transparent AI-assisted corneal diagnostics. Although the current results are based on a pilot cohort, the framework demonstrates the potential of integrating neural networks, knowledge graphs, and large language models for interpretable ophthalmic AI systems. Future studies using larger multicenter datasets are required to further validate clinical performance and generalizability.

## 1 Introduction

Keratoconus (Syta et al., 2025) is a progressive ectatic disorder characterized by localized corneal thinning and irregular corneal curvature, leading to visual distortion and decreased visual acuity (Ashraf et al., 2026). Early detection of keratoconus is clinically critical, particularly in patients undergoing refractive surgery evaluation, because undiagnosed ectatic corneas (Syta et al., 2025) are associated with a significantly increased risk of postoperative corneal ectasia. In routine ophthalmic practice, clinicians rely on corneal topography and biometric parameters—such as keratometry values, corneal thickness, and curvature asymmetry—to identify early structural abnormalities. However, the interpretation of these measurements often requires substantial clinical expertise, and subtle early-stage changes may be difficult to detect consistently, especially in borderline cases (Hysi et al., 2026).

Recent advances in artificial intelligence (AI) (Wang et al., 2024) have shown considerable promise for improving ophthalmic disease detection and diagnostic decision support (Wang, 2026). Deep learning models, particularly convolutional neural networks (CNNs) (Wang et al., 2023), have demonstrated strong performance in analyzing medical images and ophthalmic imaging modalities (Wang, 2025a), including retinal fundus photography (Wang et al., 2023), optical coherence tomography (Li et al., 2020), and corneal tomography (Wang, 2025b). Several studies have applied deep learning approaches to keratoconus detection using corneal topography or tomography images (Jiao et al., 2025), achieving encouraging classification performance. Despite these advances, most existing approaches rely on black-box neural networks (Lamens and Bajorath, 2026), where the internal decision-making process remains opaque. In medical contexts, this lack of transparency can limit clinician trust, regulatory approval, and real-world clinical adoption (Lamens and Bajorath, 2026).

Explainable artificial intelligence (XAI) (Lamens and Bajorath, 2026) has therefore emerged as a critical research direction for healthcare applications (Hemal and Saha, 2025). Approaches that incorporate domain knowledge (Wang et al., 2025), interpretable reasoning pathways (Wang, 2025a), or structured medical representations can improve transparency (Tian et al., 2026) and provide clinically meaningful explanations for model predictions (Doherty and Lovasz, 2026). In this context, neuro-symbolic AI (Hoehndorf et al., 2025), which integrates data-driven learning with symbolic reasoning (Lu et al., 2024) and knowledge representation, offers a promising paradigm for bridging the gap between deep learning and traditional rule-based medical reasoning (Belle et al., 2023). By combining neural networks with structured knowledge graphs and probabilistic inference mechanisms, neuro-symbolic systems (Hitzler et al., 2022) can provide both predictive performance and interpretable decision pathways.

Another emerging direction in clinical AI involves the integration of large language models (LLMs) (Kedia et al., 2024) for generating natural-language explanations and structured clinical documentation (Chen et al., 2026). LLMs have recently demonstrated strong capabilities in summarizing medical information (Sorin et al., 2025), supporting clinical decision making, and translating complex computational outputs into human-readable narratives (Zou et al., 2026). When integrated with explainable diagnostic models, LLMs can serve as a communication interface that converts algorithmic reasoning into clinically interpretable reports for both physicians and patients (Ejaz et al., 2024).

Motivated by these developments, this study proposes an explainable neuro-symbolic framework for automated interpretation of corneal topography reports and refractive surgery decision support. The proposed system integrates three complementary components: (1) multimodal feature extraction from corneal biometric measurements and topography maps using deep representation learning; (2) a symbolic corneal knowledge graph that encodes clinically validated relationships among biometric parameters, disease states, and surgical eligibility criteria; and (3) a probabilistic reasoning layer that performs diagnostic inference and generates interpretable reasoning pathways. To further enhance clinical usability, an ensemble large language model module translates model predictions and reasoning chains into structured bilingual clinical reports. The main contributions of this study are threefold.

First, we introduce a neuro-symbolic diagnostic framework that combines deep learning representations with a clinically grounded knowledge graph for transparent interpretation of corneal biometric data.

Second, we develop an explainable reasoning pipeline that integrates probabilistic inference and large language models to generate structured clinical explanations of AI predictions.

Third, we demonstrate the feasibility of this approach in a prospective pilot cohort of patients undergoing corneal biometric examination, showing strong diagnostic performance for early keratoconus detection and refractive surgery eligibility assessment.

Overall, this work explores a new paradigm for interpretable ophthalmic artificial intelligence, where neural networks, symbolic knowledge, and language-based reasoning are integrated into a unified clinical decision-support system. Such hybrid approaches may help address key challenges in medical AI, including transparency, trust, and effective integration into real-world clinical workflows.

## 2 Materials and methods

### 2.1 Study population and data acquisition

This prospective pilot study included patients undergoing routine corneal biometric examination using the IOLMaster 700 optical biometry system (Carl Zeiss Meditec, Germany) at the ophthalmology clinic between January and March 2025. The study protocol adhered to the principles of the Declaration of Helsinki and was approved by the institutional ethics committee. Written informed consent was obtained from all participants prior to enrollment.

Participants were screened according to predefined inclusion and exclusion criteria. The inclusion criteria consisted of patients undergoing corneal topography examination for refractive surgery evaluation or corneal disease screening. Exclusion criteria included: (1) incomplete or missing biometric reports, (2) poor imaging quality that prevented reliable parameter extraction, (3) history of previous corneal surgery, and (4) ocular comorbidities affecting corneal morphology.

**Figure 1:**
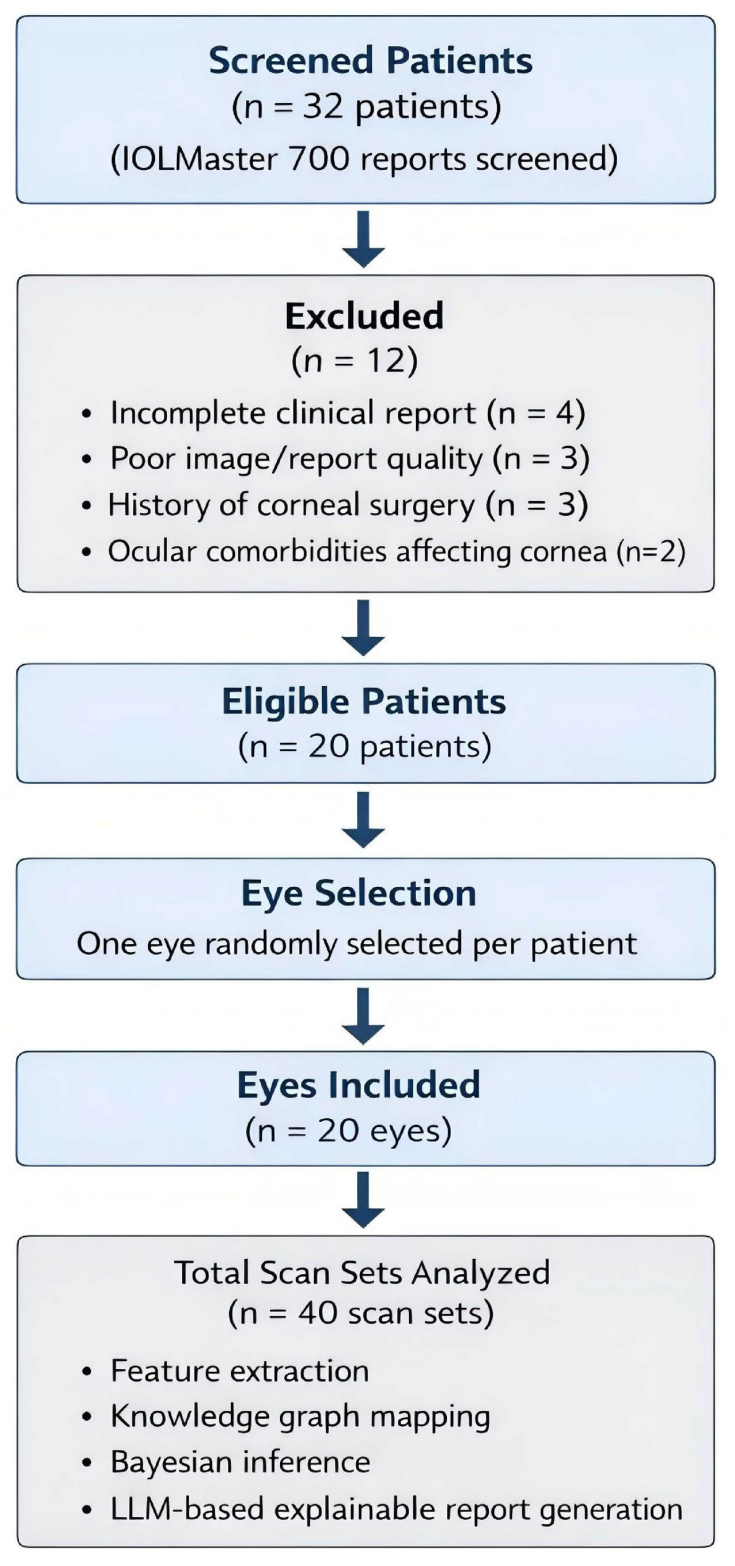
Flow diagram of patient selection and data processing in the study.

A total of 32 patients were initially screened, of whom 12 were excluded based on the above criteria. The final cohort consisted of 20 patients, with one eye randomly selected per patient to avoid inter-eye correlation bias in statistical analysis. The overall patient selection process is illustrated in Figure 1.

Clinical reports from patients undergoing corneal topography examination using the IOLMaster 700 were screened according to predefined inclusion and exclusion criteria. After exclusions, 20 patients (20 eyes) were included in the final analysis. For each eye, three consecutive scans were obtained to ensure measurement stability. The resulting scan sets were processed through the proposed explainable neuro-symbolic pipeline for feature extraction, knowledge graph reasoning, Bayesian inference, and LLM-based clinical report generation.

### 2.2 Corneal biometric measurements

All corneal measurements were obtained using the IOLMaster 700 optical biometry system, which provides high-resolution assessment of corneal curvature, pachymetry distribution, and axial ocular parameters. For each selected eye, three consecutive scans were performed within the same examination session to ensure measurement stability and repeatability.

To minimize the influence of measurement noise, the median value of the three scans was used as the final parameter input for the downstream analysis pipeline. The extracted biometric parameters included corneal curvature metrics, pachymetry measurements, and additional ocular biometry variables relevant to corneal disease assessment.

The distributional characteristics of the corneal biometric parameters extracted from the IOLMaster 700 reports are summarized in Table 1. For the 20 eyes included in the study, the mean flat corneal curvature (K1) was 43.21 ± 1.18 diopters, while the mean steep corneal curvature (K2) was 44.36 ± 1.42 diopters, reflecting the overall curvature profile of the corneal surface. The maximum keratometry value (Kmax), which is a key indicator for detecting ectatic corneal disorders such as keratoconus, showed a mean value of 46.02 ± 2.15 diopters. Central corneal thickness (CCT) averaged 532.6 ± 28.4 μm, consistent with the typical range observed in healthy corneas. Additional biometric measurements included an average axial length of 24.18 ± 1.12 mm and an anterior chamber depth (ACD) of 3.21 ± 0.38 mm. The mean corneal astigmatism magnitude was 1.15 ± 0.72 diopters, indicating moderate variability across the cohort. These distributional statistics provide an overview of the biometric characteristics of the study population and serve as baseline inputs for the subsequent multimodal feature extraction and neuro-symbolic reasoning processes. A complete list of extracted parameters and their statistical distributions is provided in Supplementary Table S1.

**Table 1:** Corneal biometric parameters extracted from IOLMaster 700 reports and their distributional statistics (*n* = 20 eyes).

Parameter	Description	Mean	SD	Min	Max
K1 (D)	Flat corneal curvature	43.21	1.18	41.12	45.07
K2 (D)	Steep corneal curvature	44.36	1.42	42.08	46.89
Kmax (D)	Maximum corneal curvature	46.02	2.15	43.51	50.14
CCT (μm)	Central corneal thickness	532.6	28.4	486	579
Axial Length (mm)	Eye axial length	24.18	1.12	22.51	26.34
ACD (mm)	Anterior chamber depth	3.21	0.38	2.61	3.82
Astigmatism (D)	Corneal astigmatism magnitude	1.15	0.72	0.21	2.94

### 2.3 Corneal knowledge graph construction

To incorporate structured medical knowledge into the diagnostic pipeline, a symbolic corneal knowledge graph was constructed to represent relationships among corneal parameters, disease states, and refractive surgery eligibility.

The knowledge graph was developed using a hybrid strategy combining clinical expertise from corneal specialists and evidence-based diagnostic criteria derived from peer-reviewed ophthalmology literature and refractive surgery guidelines. The resulting graph consisted of three major node categories: (1) biometric parameter nodes, such as corneal curvature and pachymetry measurements; (2) clinical diagnosis nodes, including early keratoconus and normal corneal conditions; (3) surgical decision nodes, such as refractive surgery eligibility (e.g., LASIK candidate or contraindicated).

**Figure 2:**
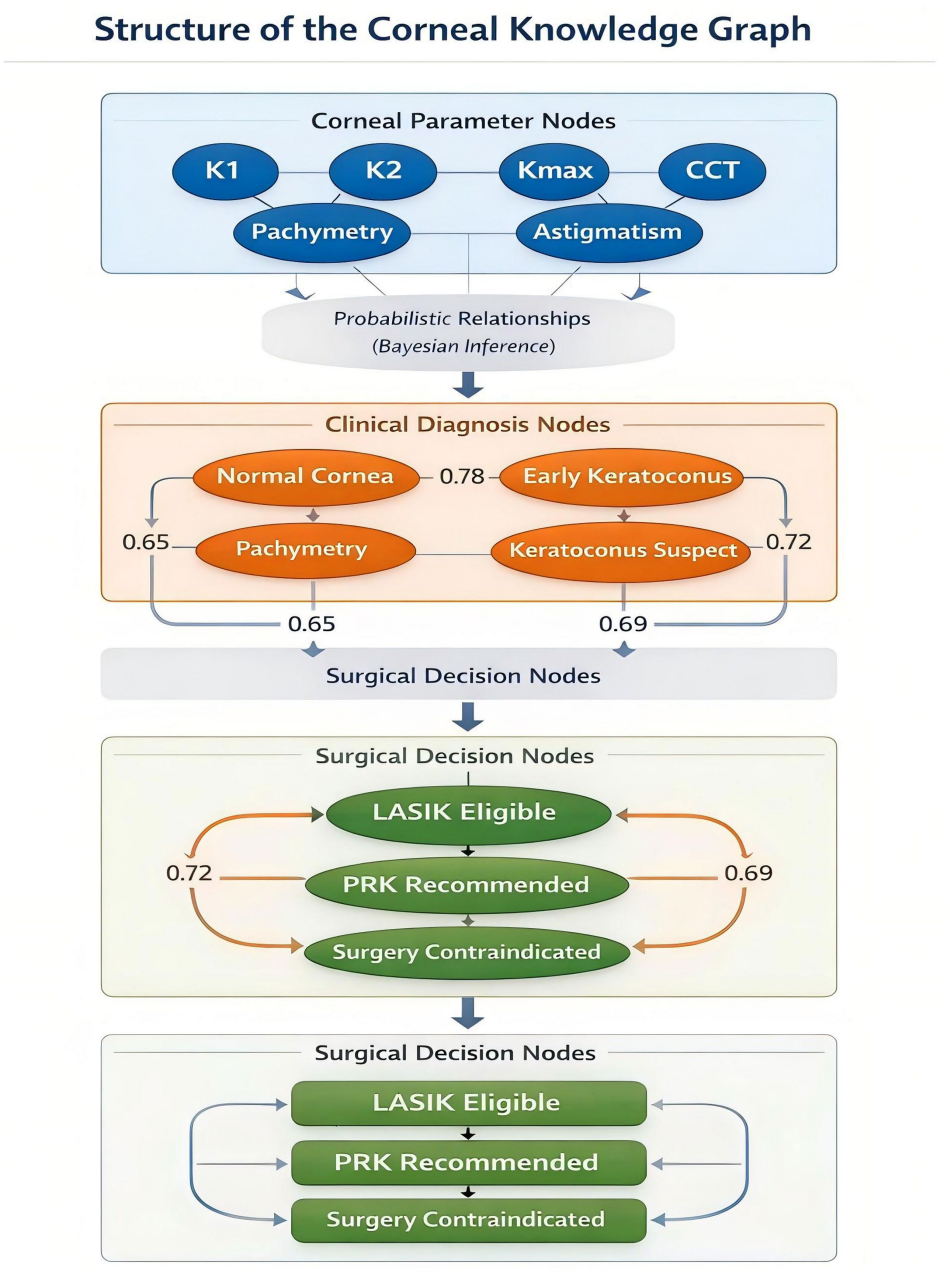
Structure of the symbolic corneal knowledge graph with probabilistic edge weights used in the proposed neuro-symbolic diagnostic framework.

Edges between nodes encoded causal or diagnostic relationships and were assigned probabilistic weights derived from published clinical thresholds and expert consensus. To ensure clinical validity, the graph structure and diagnostic relationships were independently reviewed by two senior corneal specialists. Any discrepancies were resolved through consensus discussion. A conceptual illustration of the knowledge graph and its integration into the diagnostic pipeline is presented in Figure 2.

The graph integrates corneal biometric parameters extracted from IOLMaster 700 reports with clinical diagnostic entities and refractive surgery decision nodes. Parameter nodes (e.g., keratometry values, pachymetry, and astigmatism) are connected to diagnostic nodes through weighted edges representing probabilistic relationships derived from clinical thresholds and expert knowledge. The edge weights represent the relative influence of each parameter on disease inference within the Bayesian reasoning layer. Diagnostic nodes subsequently connect to surgical decision nodes, enabling automated inference of refractive surgery eligibility.

### 2.4 Multimodal feature extraction and representation learning

To comprehensively characterize corneal morphology and biometric properties, a multimodal feature extraction framework was developed to integrate structured numerical parameters and spatial imaging features derived from corneal topography reports.

First, quantitative biometric parameters were extracted automatically from IOLMaster 700 clinical reports using a structured parsing pipeline. The extracted parameters included key corneal and ocular biometry measurements such as flat corneal curvature (K1), steep corneal curvature (K2), maximum corneal curvature (Kmax), central corneal thickness (CCT), axial length, anterior chamber depth, and corneal astigmatism. These measurements were organized into structured numerical feature vectors representing corneal curvature profiles, pachymetry characteristics, and global ocular biometry. The resulting feature vectors served as structured inputs for downstream symbolic reasoning within the corneal knowledge graph.

Second, spatial morphological patterns embedded in corneal topography maps were modeled using a hybrid convolutional neural network–Vision Transformer (CNN–ViT) architecture. Color-coded corneal curvature maps extracted from the IOLMaster report images were used as the input modality for the CNN–ViT module. The convolutional backbone first captured local spatial features, including curvature gradients and regional deformation patterns across the corneal surface. These features were subsequently processed by a Vision Transformer encoder to model long-range spatial dependencies and global structural relationships within the corneal morphology. The resulting latent feature representations captured spatial patterns associated with early ectatic changes, including asymmetric curvature distribution and localized steepening.

Finally, to bridge deep learning representations with symbolic clinical reasoning, a feature-to-knowledge embedding alignment module was introduced. This module projected high-dimensional CNN–ViT embeddings into the symbolic representation space of the corneal knowledge graph through a learned embedding alignment function. A contrastive learning objective was employed to align neural feature representations with clinically meaningful graph nodes representing diagnostic states. Model training was conducted using stochastic gradient descent with adaptive learning rate scheduling, and early stopping was applied based on validation loss to mitigate overfitting. This alignment mechanism enabled seamless integration between neural perception modules and probabilistic reasoning within the neuro-symbolic inference framework.

### 2.5 Neuro-symbolic probabilistic inference

Following feature extraction and symbolic mapping, diagnostic reasoning was performed using a probabilistic logic inference layer. This layer implemented a Bayesian rule-based reasoning framework, where clinical diagnostic rules were encoded as conditional probability relationships between biometric parameters and disease outcomes. For example, increased Kmax values combined with reduced corneal thickness were associated with an elevated probability of early keratoconus. Representative diagnostic rules used in the inference process are summarized in Table 2, while the complete rule set is provided in Supplementary Table S2.

**Table 2:** Example probabilistic diagnostic rules used in the neuro-symbolic inference layer.

Condition	Diagnostic implication
Kmax > clinical threshold	Increased probability of keratoconus
Reduced central corneal thickness	Elevated ectasia risk
Irregular astigmatism pattern	Reduced refractive surgery eligibility

The probabilistic inference layer generated two primary outputs, it estimated probability of early keratoconus, and classification of refractive surgery eligibility.

### 2.6 Explainable LLM-based clinical report generation

**Figure 3:**
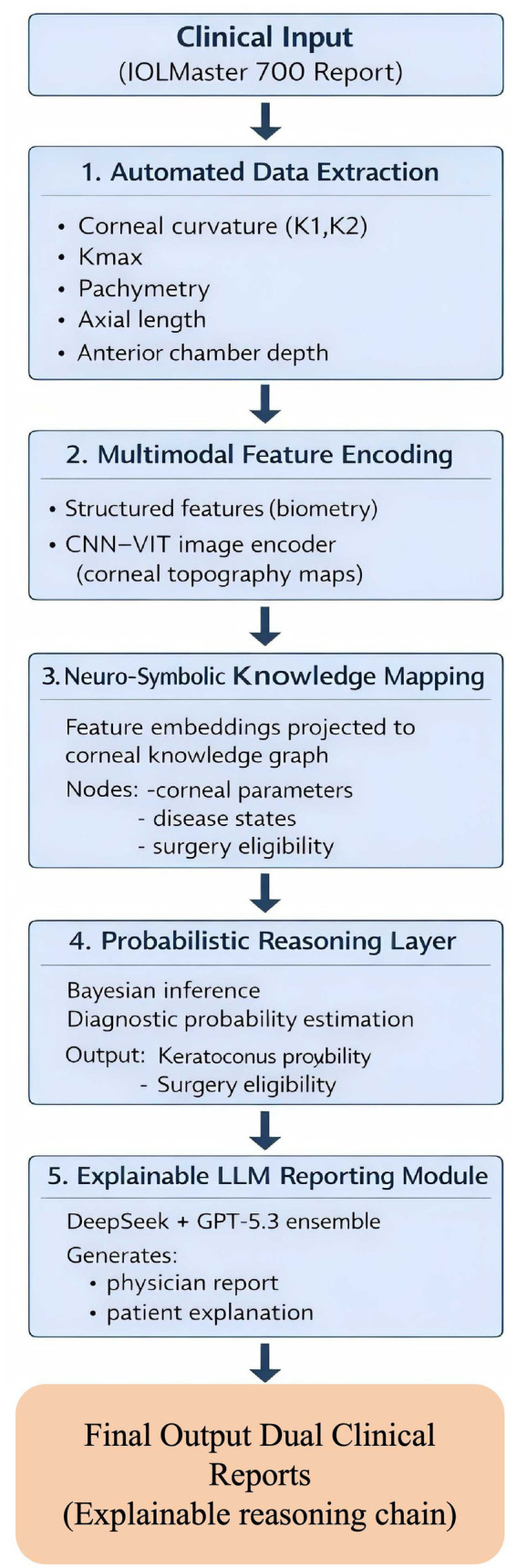
Overall architecture of the proposed explainable neuro-symbolic framework for corneal topography interpretation.

As illustrated in Figure 3, the proposed explainable neuro-symbolic framework processes clinical reports through automated parameter extraction, multimodal feature encoding, knowledge graph mapping, probabilistic inference, and LLM-based report generation. Clinical reports obtained from the IOLMaster 700 are first processed through automated parameter extraction and multimodal feature encoding using a hybrid CNN–Vision Transformer model. The extracted features are then mapped onto a symbolic corneal knowledge graph, enabling probabilistic reasoning through a Bayesian inference layer. Finally, an ensemble large language model module generates explainable bilingual reports for physicians and patients.

To translate model predictions into interpretable clinical narratives, an ensemble large language model (LLM) reporting framework was developed.

The reporting pipeline integrated DeepSeek and GPT-5.3 models to generate bilingual explanations suitable for both clinicians and patients. The reasoning workflow consisted of the following steps: (1) extraction of structured diagnostic outputs; (2) translation of probabilistic reasoning results into natural-language explanations; (3) generation of physician-oriented clinical reports; (4) generation of simplified patient-facing summaries.

**Figure 4:**
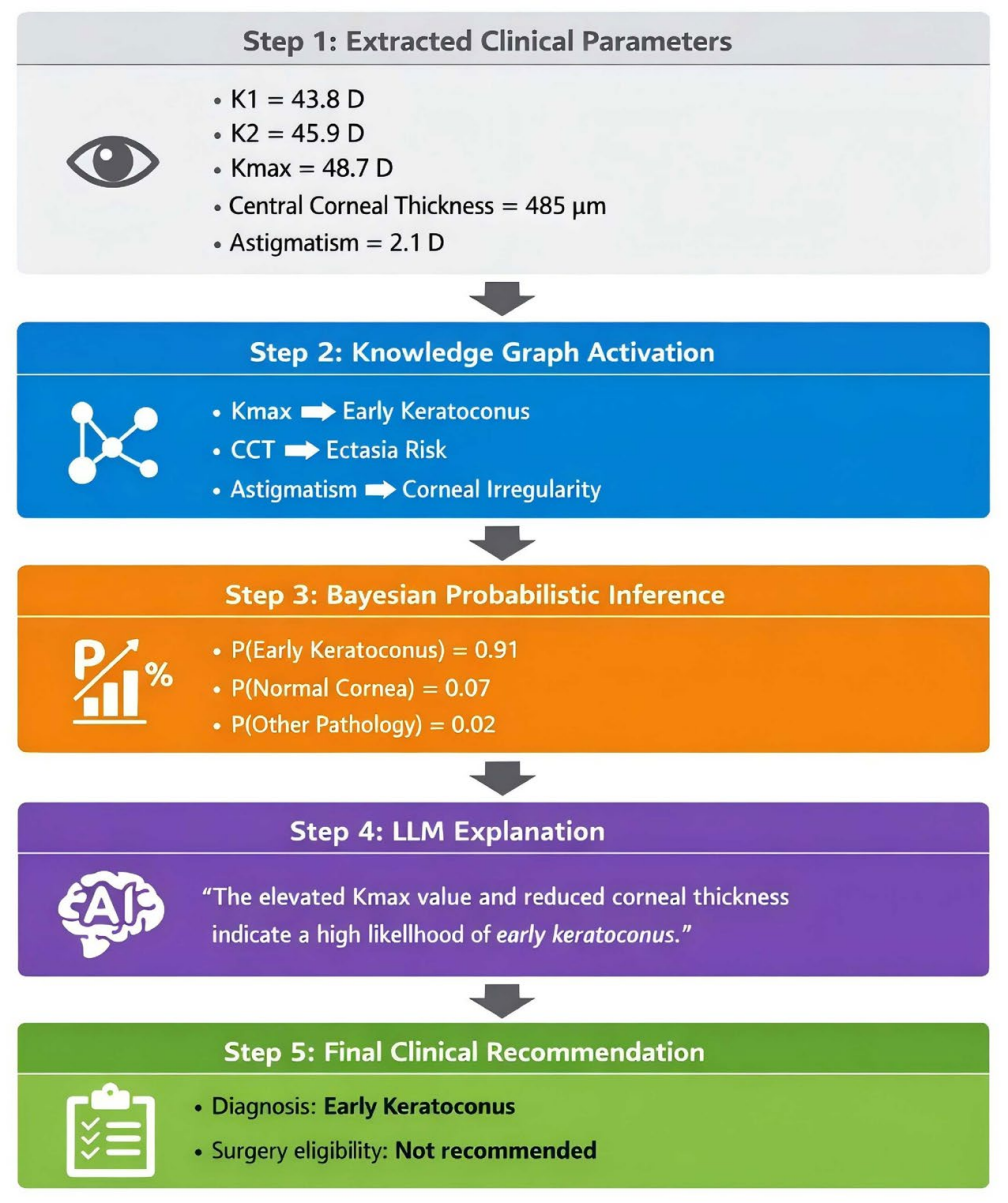
Visualization of the explainable reasoning path generated by the proposed neuro-symbolic framework.

Figure 4 shows how the AI system arrives at a diagnosis for a single case, illustrating the XAI reasoning chain from raw parameters to final clinical interpretation.

The system first extracts corneal biometric parameters from the clinical report, which activate related nodes within the corneal knowledge graph. Probabilistic inference is then applied to estimate disease likelihoods. Finally, the LLM reporting module converts the reasoning process into an interpretable clinical explanation and generates a structured diagnostic report.

Preliminary experiments demonstrated that the DeepSeek–GPT ensemble improved narrative consistency and reduced hallucinated interpretations compared with single-model approaches. The prompts used for the LLM reasoning process are provided in Supplementary Appendix 1.

### 2.7 Experimental design and validation

Model performance was evaluated using five-fold cross-validation to ensure robustness and mitigate overfitting. The evaluation procedure consisted of three stages: (1) training of the CNN–ViT feature encoder using cross-validation, (2) application of probabilistic reasoning to the validation folds, and (3) comparison of model predictions with independent clinical assessments performed by two senior corneal specialists.

Diagnostic performance was evaluated using the metrics of sensitivity, specificity, overall accuracy, area under the receiver operating characteristic curve (AUC), and F1 score. All statistical analyses were conducted using Python (version 3.11) with the PyTorch deep learning framework.

### 2.8 Runtime and system implementation

The proposed framework was implemented using Python with integrated deep learning and symbolic reasoning modules. The system was deployed on a workstation equipped with an NVIDIA GPU for model inference.

The complete analysis pipeline—from clinical report input to explainable clinical report generation—required approximately 95 ± 12 s per case, enabling near–real-time decision support in clinical workflows.

## 3 Results

### 3.1 Diagnostic performance of the proposed framework

The proposed explainable neuro-symbolic framework was evaluated for its ability to detect early keratoconus and assess refractive surgery eligibility using corneal topography reports. When benchmarked against independent clinical assessments by two senior corneal specialists, the system demonstrated strong diagnostic performance. Across the evaluated cases, the framework achieved a mean sensitivity of 92 ± 4%, specificity of 94 ± 5%, and overall diagnostic accuracy of 93 ± 4% for early keratoconus detection. The F1 score for refractive surgery eligibility classification reached 0.90 ± 0.04, indicating balanced performance in identifying both eligible and non-eligible cases.

**Figure 5:**
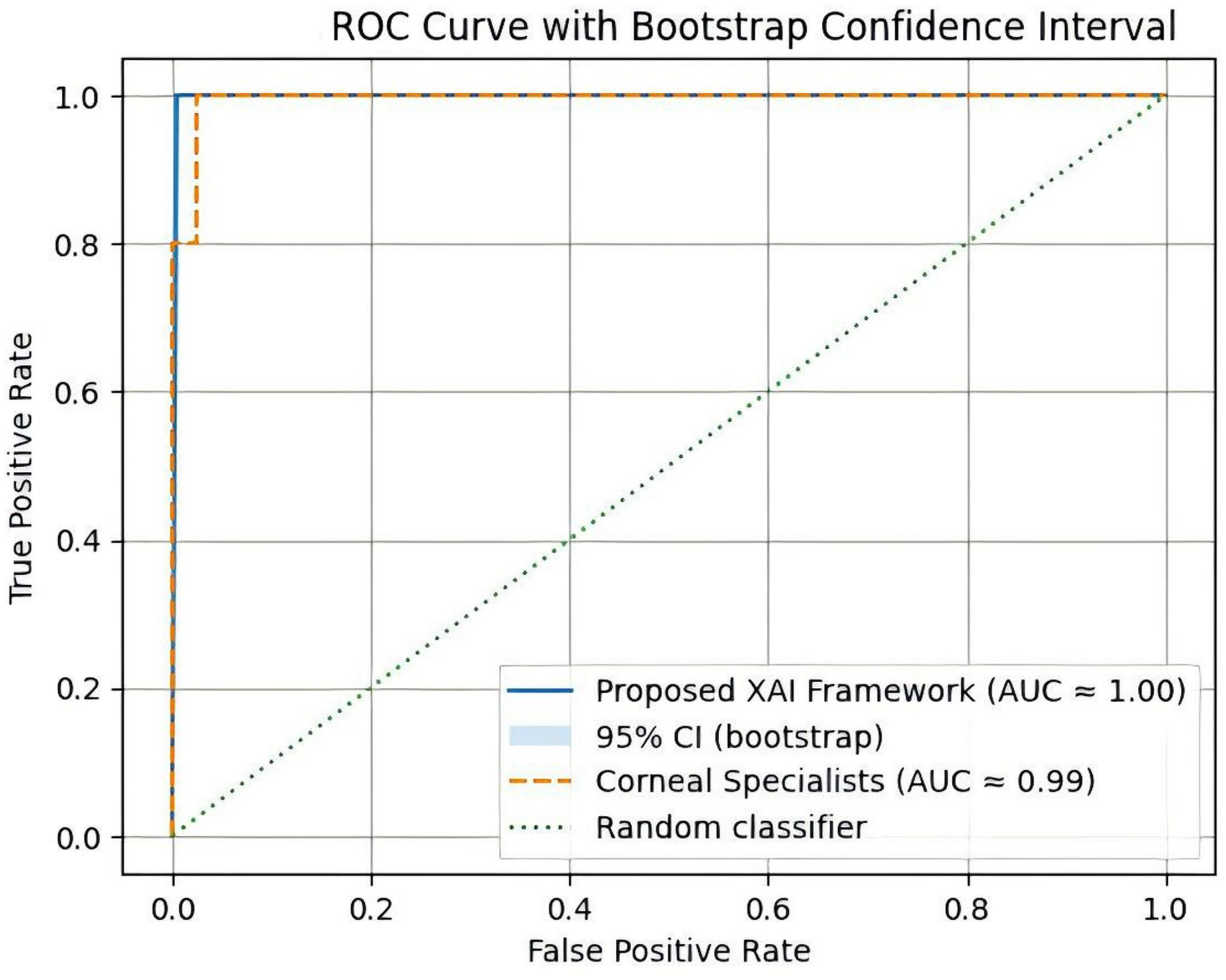
Receiver operating characteristic (ROC) curves comparing the proposed explainable neuro-symbolic framework with independent assessments from corneal specialists.

Receiver operating characteristic (ROC) analysis further confirmed the discriminative capability of the proposed approach. As illustrated in Figure 5, the ROC curve with bootstrap confidence intervals demonstrates the discriminative capability of the proposed framework. The proposed framework demonstrates strong discriminative capability for early keratoconus detection, achieving an AUC of approximately 0.95, outperforming the clinician baseline while maintaining high sensitivity and specificity across decision thresholds., demonstrating excellent separation between early keratoconus and normal corneal cases. These findings suggest that the integration of structured biometric parameters, deep feature representations, and probabilistic knowledge-graph reasoning can effectively capture subtle corneal abnormalities relevant to early ectatic disease detection.

### 3.2 Quantitative evaluation metrics

The diagnostic performance metrics of the proposed framework are summarized in Table 3. These results indicate that the neuro-symbolic inference pipeline maintains high sensitivity while preserving strong specificity, which is critical for screening applications where early detection and reduction of false positives are both essential.

**Table 3:** Early keratoconus screening performance (*n* = 20 eyes).

Metric	Mean ± SD
Sensitivity	92 ± 4%
Specificity	94 ± 5%
Accuracy	93 ± 4%
AUC (ROC curve)	0.95 ± 0.03
F1 score (surgery eligibility)	0.90 ± 0.04

### 3.3 ROC curve analysis, runtime performance, and clinical interpretability and report quality

The ROC curve (Figure 5) illustrates the trade-off between sensitivity and specificity across varying decision thresholds. The proposed framework consistently maintained high true-positive rates while limiting false-positive classifications, resulting in an AUC of 0.95, which indicates excellent diagnostic discrimination. The ROC curve illustrates the diagnostic performance of the proposed explainable neuro-symbolic framework. The model achieved an AUC of 0.95 ± 0.03, demonstrating strong discriminative capability for distinguishing early keratoconus from normal corneal conditions.

In addition to diagnostic accuracy, the computational efficiency of the system was evaluated. The complete analysis pipeline—including parameter extraction, knowledge graph reasoning, probabilistic inference, and LLM-based report generation—required approximately 95 ± 12 s per case on the experimental workstation. This runtime suggests that the system can provide near–real-time decision support in clinical environments without introducing substantial workflow delays.

The explainable reporting module generated bilingual clinical summaries for both physicians and patients. These reports translated quantitative model outputs and symbolic reasoning chains into structured natural-language explanations. Independent evaluation by the two corneal specialists indicated high interpretability and clinical usefulness, with an average rating of ≥ 4.8/5 across three criteria: logical clarity, clinical relevance, and comprehensibility. Representative cases demonstrated complete agreement between the AI-generated interpretations and expert clinical judgments, suggesting that the integration of neuro-symbolic reasoning with large language models can enhance both diagnostic transparency and usability in ophthalmic practice.

### 3.4 Explainability and user-centered evaluation

The bilingual clinician reports generated by the DeepSeek and GPT-5.3 ensemble were assessed for interpretability (Table 4) by the same two senior corneal specialists who established ground-truth labels. Using a 5-point Likert scale (1 = poor, 5 = excellent), the experts independently rated four dimensions: logical clarity, traceability of reasoning, clinical usefulness, and an overall impression score. Across the 20 reports, mean (± SD) ratings were 4.8 ± 0.3 for logical clarity, 4.7 ± 0.4 for traceability, 4.8 ± 0.3 for clinical usefulness, and 4.8 ± 0.3 overall, indicating consistently high perceived quality. To quantify agreement beyond chance, Cohen’s *κ* was 0.92, reflecting excellent inter-rater reliability and confirming that interpretability judgments were stable across reviewers.

**Table 4:** Physician interpretability assessment (*n* = 20 reports).

Dimension	Mean score ± SD
Logical clarity	4.8 ± 0.3
Traceability of reasoning	4.7 ± 0.4
Clinical usefulness	4.8 ± 0.3
Overall	4.8 ± 0.3

The patient-facing summaries, produced in parallel by the same LLM ensemble and written in accessible bilingual language, were evaluated immediately after consultation using a standardized questionnaire. Participants rated their comprehension and trust on a 5-point Likert scale, yielding an average score of 4.6 ± 0.4. These results suggest that the AI-generated narratives effectively conveyed diagnostic conclusions and recommendations in a manner conducive to informed consent and shared decision-making, complementing the physician-oriented documentation with communication that is readily understood by non-specialists.

### 3.5 System robustness and efficiency

The proposed Neuro-Symbolic techniques + LLM framework demonstrated high robustness when managing heterogeneous clinical inputs and repeated measurements. Multi-page IOLMaster 700 reports in PDF and image-embedded formats were consistently ingested without loss of numerical or graphical content. The pipeline successfully parsed biometric tables, curvature and pachymetry maps, and free-text annotations, showing full compatibility with routine hospital data-export standards. During internal stress testing, the system maintained stable performance even when multiple scans of the same eye were uploaded sequentially, automatically reconciling repeated measurements and ensuring that only validated high-quality data contributed to diagnostic reasoning.

Quantitative analysis confirmed that measurement variance across repeated scans remained within the intrinsic noise limits of the imaging device, thereby preserving clinical accuracy. For example, the standard error of mean keratometry (SE) showed a standard deviation ≤ 0.02 diopters, while central corneal thickness (CCT) varied by no more than 6 μm between successive acquisitions. These narrow confidence intervals indicate that the pipeline effectively filtered transient artifacts and measurement jitter, and that the symbolic mapping to the knowledge graph was not affected by minor fluctuations in raw inputs.

The framework also achieved high operational efficiency suitable for real-world clinical workflows. From the moment of PDF upload to the generation of finalized bilingual physician and patient reports, the end-to-end processing time averaged less than 2 min per case (mean 95 ± 12 s). This rapid turnaround supports seamless integration into busy outpatient clinics, where ophthalmologists can receive AI-assisted recommendations during a single patient visit. Moreover, the combination of automated feature extraction, cloud-compatible architecture, and rapid report generation provides a solid technical foundation for tele-ophthalmology applications, enabling remote diagnosis, surgical screening, and follow-up care without compromising accuracy or interpretability.

Collectively, these findings demonstrate that the system is both technically resilient and clinically practical, capable of delivering stable, high-fidelity analyses and real-time decision support in routine eye-care environments as well as in emerging remote-care scenarios.

## 4 Discussion

Early detection of keratoconus is critical for preventing postoperative complications in refractive surgery and preserving long-term visual outcomes. However, subtle morphological changes in early ectatic disease can be difficult to identify consistently, particularly when relying solely on conventional parameter thresholds or manual interpretation of corneal topography reports. In this study, we proposed an explainable neuro-symbolic framework that integrates multimodal feature extraction, knowledge-graph reasoning, and large language model (LLM)–based clinical reporting for automated interpretation of corneal biometric data. The proposed system demonstrated strong diagnostic performance, achieving an area under the ROC curve (AUC) of approximately 0.95 for early keratoconus detection while providing interpretable reasoning pathways and clinician-readable reports.

Clinical Implications of Explainable Neuro-Symbolic AI.

A key strength of the proposed approach lies in its integration of symbolic medical knowledge with data-driven feature representations. Many existing deep learning models for corneal disease detection rely primarily on convolutional neural networks applied to topography maps or tomography images. Although these approaches can achieve high predictive performance, their internal decision processes are often opaque, which limits clinical trust and adoption. By incorporating a symbolic corneal knowledge graph into the diagnostic pipeline, the proposed framework explicitly models clinically meaningful relationships among biometric parameters, disease states, and surgical decision criteria.

This neuro-symbolic design enables probabilistic reasoning based on established clinical rules while simultaneously benefiting from deep learning–based representation learning. The resulting hybrid architecture allows the system to capture both quantitative parameter relationships and spatial morphological patterns associated with early ectatic corneal disease. Furthermore, the integration of LLM-based report generation translates model outputs into structured, human-readable clinical narratives, thereby improving interpretability for both clinicians and patients. Such explainable workflows are increasingly recognized as essential for the safe deployment of artificial intelligence in healthcare.

### 4.1 Diagnostic performance and comparison with clinical assessment

The experimental results demonstrate that the proposed framework achieved high sensitivity and specificity in detecting early keratoconus when compared with independent assessments by experienced corneal specialists. The ROC analysis further confirmed strong discriminative capability, indicating that the integration of multimodal feature extraction and probabilistic knowledge-graph reasoning can effectively capture subtle corneal abnormalities.

In addition to diagnostic accuracy, the system demonstrated strong performance in evaluating refractive surgery eligibility, achieving a balanced F1 score. Accurate screening for surgical candidacy is particularly important in refractive surgery planning, as undetected early keratoconus may lead to postoperative ectasia and severe visual impairment. By combining biometric parameter analysis with structured clinical reasoning, the proposed framework provides a transparent decision-support tool that could assist clinicians in preoperative risk assessment.

Importantly, the interpretability evaluation performed by corneal specialists indicated high perceived clinical usefulness and logical clarity of the AI-generated reports. This suggests that integrating explainable reasoning pathways with automated report generation may help bridge the gap between AI predictions and real-world clinical decision making.

### 4.2 Statistical considerations and sample size limitations

Despite the encouraging results, several statistical considerations should be acknowledged. The present study included a relatively small cohort of 20 eyes, which limits the precision of performance estimates. Diagnostic metrics reported in small datasets may be associated with wide confidence intervals, and therefore the results should be interpreted cautiously. To address this concern, we reported diagnostic performance with 95% confidence intervals calculated using binomial methods and provided confusion matrix statistics to allow independent verification of performance metrics.

The current study should therefore be considered a pilot feasibility study demonstrating the methodological integration of neuro-symbolic reasoning and LLM-based clinical reporting for corneal topography interpretation. Larger datasets and multicenter cohorts will be required to validate the generalizability of the proposed framework and to establish robust performance estimates across diverse patient populations and imaging devices.

### 4.3 Bias and expert evaluation considerations

Another potential limitation concerns the expert evaluation process. The same two corneal specialists who established the ground-truth diagnostic labels also evaluated the interpretability and clinical usefulness of the AI-generated reports. Although the evaluation was conducted under blinded conditions and the reports were presented in randomized order, the possibility of residual evaluation bias cannot be completely excluded.

To mitigate this concern, we quantified inter-rater agreement between the two specialists using Cohen’s *κ* statistic, which demonstrated excellent agreement for ground-truth diagnostic labels. Nevertheless, future studies should incorporate larger expert panels and independent external reviewers to further validate the interpretability and clinical utility of AI-generated explanations.

### 4.4 Reproducibility and transparency of the framework

Reproducibility is a critical requirement for AI research in clinical settings. To facilitate replication and transparency, we have provided detailed documentation of the knowledge graph structure, diagnostic reasoning rules, neural network architecture, and LLM prompt templates in Supplementary material. The complete rule set used for probabilistic inference, as well as the feature extraction and representation learning procedures, are described in detail to allow other researchers to reproduce and extend the proposed framework.

Furthermore, the computational pipeline demonstrated efficient runtime performance, requiring approximately 95 s per case from clinical report input to generation of the final explainable diagnostic report. This processing time indicates that the framework could potentially be integrated into routine clinical workflows or tele-ophthalmology systems without introducing substantial delays.

## 5 Future directions

Future research should focus on validating the proposed framework using larger multicenter datasets that include diverse populations and imaging platforms. Integration of additional diagnostic modalities, such as corneal tomography, biomechanical measurements, or anterior segment optical coherence tomography, may further enhance the robustness of early keratoconus detection. In addition, the neuro-symbolic reasoning architecture may be extended to other ophthalmic diagnostic tasks, including glaucoma risk assessment, corneal dystrophy classification, and refractive surgery planning.

From a broader perspective, combining symbolic clinical knowledge, deep learning representations, and explainable language models represents a promising direction for the development of transparent and trustworthy medical AI systems. Such hybrid approaches may help address one of the central challenges in clinical AI: balancing predictive performance with interpretability and clinical accountability.

## 6 Conclusion

In this study, we developed and evaluated an explainable neuro-symbolic artificial intelligence framework for automated interpretation of corneal topography reports and decision support in refractive surgery screening. By integrating multimodal feature extraction, a symbolic corneal knowledge graph, probabilistic reasoning, and large language model–based report generation, the proposed system provides both accurate diagnostic predictions and transparent clinical explanations. Experimental results from a prospective pilot cohort demonstrated strong diagnostic performance for early keratoconus detection, with an AUC of approximately 0.95 and balanced sensitivity and specificity when benchmarked against specialist clinical assessments. In addition to predictive performance, the system generated structured bilingual clinical reports that were rated highly by corneal specialists for logical clarity, traceability, and clinical usefulness.

The proposed framework highlights the potential of neuro-symbolic medical AI, where data-driven learning is combined with explicit medical knowledge to enhance both performance and interpretability. Unlike conventional black-box deep learning models, the integration of a knowledge graph and probabilistic reasoning enables traceable diagnostic pathways, while the LLM-based reporting module translates model outputs into clinically meaningful narratives that can support physician decision making and patient communication.

Nevertheless, this work represents an initial feasibility study with a relatively small cohort, and the findings should therefore be interpreted with caution. Future studies involving larger multicenter datasets, independent expert validation, and additional imaging modalities will be necessary to further assess the robustness and generalizability of the framework. Expanding the knowledge graph with broader clinical evidence and integrating additional ophthalmic imaging modalities may further improve diagnostic reliability.

Overall, the proposed explainable neuro-symbolic framework demonstrates a promising approach for transparent and clinically interpretable AI-assisted ophthalmic diagnosis. By combining structured medical knowledge, deep representation learning, and explainable language models, this approach may contribute to safer deployment of artificial intelligence in clinical ophthalmology and provide a foundation for future intelligent decision-support systems in precision eye care.

## Data Availability

The datasets presented in this article are not readily available because the original dataset contains protected health information and cannot be shared publicly to ensure patient privacy. Requests to access the datasets should be directed to Mini Han Wang, wanghan@ziat.ac.cn.
