# Supplementary material for: Explainable neuro-symbolic artificial intelligence for automated interpretation of corneal topography and early keratoconus detection
Source: Front Artif Intell. 2026 Apr 13;9:1713747. doi: 10.3389/frai.2026.1713747 (PMC13111306; doi:10.3389/frai.2026.1713747)
Supplement: Supplementary file 1 [file Data_Sheet_1.docx]

**Supplementary Appendix A**

Large Language Model Prompts Used in the Explainable Reporting Module

To ensure reproducibility and transparency, the prompts used for the large language model reasoning process are provided below.

| Prompt A1: Clinical reasoning prompt |
| --- |
| Input: Corneal biometric parameters extracted from clinical report.  Task: Analyze the parameters using established ophthalmic diagnostic criteria.  Identify whether the pattern suggests normal corneal morphology or possible early keratoconus.  Explain the reasoning process step-by-step.  Output:  1. Key abnormal parameters  2. Clinical interpretation  3. Diagnostic conclusion |

| Prompt A2: Physician report generation prompt |
| --- |
| Generate a clinical report summarizing the diagnostic findings based on the model outputs.  Requirements:  - Use professional ophthalmology terminology  - Provide a clear diagnostic interpretation  - Explain the reasoning chain linking parameters to diagnosis |

| Prompt A3: Patient explanation prompt |
| --- |
| Rewrite the clinical findings in language understandable to a patient.  Requirements:  - Avoid complex medical terminology  - Clearly explain the meaning of the findings  - Provide recommendations for follow-up or further examination |
