# Supplementary material for: Explainable neuro-symbolic artificial intelligence for automated interpretation of corneal topography and early keratoconus detection
Source: Front Artif Intell. 2026 Apr 13;9:1713747. doi: 10.3389/frai.2026.1713747 (PMC13111306; doi:10.3389/frai.2026.1713747)
Supplement: Supplementary file 2 [file Table_1.docx]

## Supplementary Table S1

### Complete list of corneal biometric parameters extracted from IOLMaster 700 reports

This table summarizes the quantitative parameters automatically extracted from the IOLMaster 700 reports and used in the proposed neuro-symbolic diagnostic pipeline.These parameters were used as structured inputs for both the symbolic reasoning module and the deep feature alignment process.

| Parameter | Unit | Description | Mean | SD | Min | Max |
| --- | --- | --- | --- | --- | --- | --- |
| K1 | Diopters (D) | Flat corneal curvature | 42.8 | 1.3 | 40.5 | 45.2 |
| K2 | Diopters (D) | Steep corneal curvature | 44.1 | 1.6 | 41 | 47.8 |
| Kmax | Diopters (D) | Maximum corneal curvature | 46.3 | 2.1 | 42.2 | 50.5 |
| CCT | µm | Central corneal thickness | 521 | 32 | 470 | 580 |
| Astigmatism | Diopters (D) | Corneal astigmatism magnitude | 1.8 | 0.7 | 0.4 | 3.2 |
| Axial Length | mm | Eye axial length | 24.3 | 1.1 | 22.5 | 26.2 |
| ACD | mm | Anterior chamber depth | 3.1 | 0.4 | 2.6 | 3.8 |
| Pachymetry Min | µm | Minimum corneal thickness | 498 | 35 | 440 | 560 |
