# Supplementary material for: Explainable neuro-symbolic artificial intelligence for automated interpretation of corneal topography and early keratoconus detection
Source: Front Artif Intell. 2026 Apr 13;9:1713747. doi: 10.3389/frai.2026.1713747 (PMC13111306; doi:10.3389/frai.2026.1713747)
Supplement: Supplementary file 3 [file Table_2.docx]

## Supplementary Table S2

Diagnostic rules used in the probabilistic inference layer

The neuro-symbolic reasoning module applies a set of probabilistic clinical rules derived from published diagnostic criteria and expert consensus. Representative rules are summarized below. Each rule contributes to the Bayesian inference process by adjusting the posterior probability of the corresponding diagnostic node within the corneal knowledge graph. Each rule contributes to the Bayesian inference process by adjusting the posterior probability of the corresponding diagnostic node within the corneal knowledge graph.

| Rule ID | Condition | Diagnostic Implication |
| --- | --- | --- |
| R1 | Kmax > 47 D | Increased probability of keratoconus |
| R2 | CCT < 500 µm | Elevated ectatic disease risk |
| R3 | Kmax > 48 D AND CCT < 500 µm | High probability of early keratoconus |
| R4 | Astigmatism > 2.0 D | Increased corneal irregularity risk |
| R5 | CCT < 480 µm | Reduced refractive surgery eligibility |
| R6 | Normal Kmax AND normal pachymetry | Normal corneal morphology |
